# Genome-Wide Analysis of the miRNA–mRNAs Network Involved in Cold Tolerance in *Populus simonii* × *P. nigra*

**DOI:** 10.3390/genes10060430

**Published:** 2019-06-05

**Authors:** Bo Zhou, Yutong Kang, Jingtong Leng, Qijiang Xu

**Affiliations:** 1 State Key Laboratory of Tree Genetics and Breeding, Northeast Forestry University, 26 Hexing Road, Harbin 150040, China; 2 College of Life Science, Northeast Forestry University, 26 Hexing Road, Harbin 150040, China; amazingkk@sina.cn (Y.K.); lengjt558@163.com (J.L.)

**Keywords:** *Populus simonii* × *P. nigra*, cold induction, microRNAs, transcriptome

## Abstract

Background: Cold tolerance is important for plants’ geographical distribution and survival in extreme seasonal variations of climate. However, *Populus simonii* × *P. nigra* shows wide adaptability and strong cold resistance. Transcriptional and post-transcriptional regulation of cold-responsive genes is crucial for cold tolerance in plants. To understand the roles of regulatory RNAs under cold induction in *Populus simonii* × *P. nigra*, we constructed cDNA and small RNA libraries from leaf buds treated or not with −4 °C for 8 h for analysis. Results: Through high-throughput sequencing and differential expression analysis, 61 miRNAs and 1229 DEGs were identified under cold induction condition in *Populus simonii* × *P. nigra.* The result showed that miR167a, miR1450, miR319a, miR395b, miR393a-5p, miR408-5p, and miR168a-5p were downregulated, whereas transcription level of miR172a increased under the cold treatment. Thirty-one phased-siRNA were also obtained (reads ≥ 4) and some of them proceeded from TAS3 loci. Analysis of the differentially expressed genes (DEGs) showed that transcription factor genes such as Cluster-15451.2 (putative MYB), Cluster-16493.29872 (putative bZIP), Cluster-16493.29175 (putative SBP), and Cluster-1378.1 (putative ARF) were differentially expressed in cold treated and untreated plantlets of *Populus simonii* × *P. nigra.* Integrated analysis of miRNAs and transcriptome showed miR319, miR159, miR167, miR395, miR390, and miR172 and their target genes, including MYB, SBP, bZIP, ARF, LHW, and ATL, were predicted to be involved in ARF pathway, SPL pathway, DnaJ related photosystem II, and LRR receptor kinase, and many of them are known to resist chilling injury. Particularly, a sophisticated regulatory model including miRNAs, phasiRNAs, and targets of them was set up. Conclusions: Integrated analysis of miRNAs and transcriptome uncovered the complicated regulation of the tolerance of cold in *Populus simonii* × *P. nigra.* MiRNAs, phasiRNAs, and gene-encoded transcription factors were characterized at a whole genome level and their expression patterns were proved to be complementary. This work lays a foundation for further research of the pathway of sRNAs and regulatory factors involved in cold tolerance.

## 1. Background

Plants have evolved sophisticated mechanisms to adapt various abiotic injuries such as heat, drought, salinity, and low temperature during their life cycle [1]. Cold injury, including chilling (<20 °C) and frost (<0 °C) is one of the key environmental factors in cold temperate zones that affect plant development and growth [2] and decide their geographical distribution. Low temperature can lead to the decrease of photosynthesis rate, the accumulation of reactive oxygen species (ROS), reduced nutrient absorption, disrupted membrane transport, and various forms of physiological damage. Plant cold-tolerance depends on their ability to regulate gene expression to control their physiology, metabolism, and growth [3]. Besides transcriptional regulation, post-transcription regulation is also popular in plants to control their gene expression during cold induction. MicroRNAs (miRNAs) are small noncoding RNAs that have been proved to play key roles in plant development, signal transduction, metabolism, and response to environmental injury [4,5,6].

miRNAs are 20–24 nt in length and mainly originate from the pre-existing MIR genes and protein-coding genes [7]. In plants, stem-loop or hairpin structure RNAs are recognized by RNase III-like enzymes (Dicer-Like, DCL) and processed into small RNAs, and then incorporated into an RNA-induced silencing complex (RISC). Finally, the mature miRNAs bind their target mRNAs through imperfect sequence complementarity to negatively regulate the expression of target genes [8,9]. Nowadays, a large number of miRNAs have been identified that perform regulatory functions in different plant species and are deposited in miRBase [10]. Recently many miRNAs have been reported to be involved in cold resistance responses in *Arabidopsis* [11,12], poplar [2,13], rice [14], tea plant [15], eggplant [16], and wheat [17]. In *Arabidopsis*, the expression levels of miR156, miR159, miR164, miR168, miR172, miR165/166, miR169, miR393, and miR319c are related to cold response [12,18,19], and the upregulated miRNAs are approximately 17% of *Arabidopsis* miRNAs [11]. However, in rice, 18 cold responsive miRNAs were identified, and most were downregulated [14]. Also, among 30 cold responsive miRNAs, 21 were validated to be downregulated in *Populus tomentosa* [2]. In a cold-tolerant tea plant cultivar, 31 upregulated miRNAs and 43 downregulated miRNAs were identified, while in a cold-sensitive tea plant cultivar, 46 upregulated miRNA and 45 downregulated miRNAs were evaluated in response to cold resistance [15]. In eggplant, 56 downregulated miRNAs and 28 upregulated miRNAs were also identified [16]. Moreover, 39 miRNAs were differentially expressed under cold treatment in wheat and among them, 23 were downregulated [20]. Cold injury negatively affects plant growth and development through cold-responsive gene regulatory networks. Apart from miRNAs, the activation of C-repeat binding factor (CBF) and cold-regulated (COR) genes have also been characterized to respond to cold resistance [21,22]. C-repeat binding factor 3(AtCBF3) and cold-regulated 15A (AtCOR15A) has been reported to enhance cold tolerance of transgenic eggplants [23].

*Populus simonii* × *P. nigra*, the hybrid crossed by *P. simonii* and *P. nigra*, is a fast-growing species widely distributed in North China, and can adapt to certain levels of cold climate. Due to its broad adaptability and resistance to abiotic injury, the poplar has been planted in commercial forests and for landscape enhancement in North China. However, in Heilongjiang province, the northeast of China, *Populus simonii* × *P. nigra* sometimes suffer from low temperature in spring. Therefore, the molecular mechanism that regulates plant responses in cold tolerance of *Populus simonii* × *P. nigra* remains to be understood. Based on the study of cold-related genes in *Arabidopsis* and other plant species, including Chinese white poplar (*Populus tomentosa*) [2], further research is necessary to identify miRNAs and their target genes involved in cold tolerance in *Populus simonii* × *P. nigra* through transcriptome and small RNA sequencing analysis.

To further explore the miRNAs and COR genes in response to cold resistance and understand the complex molecular regulation mechanism of cold tolerance in *Populus simonii* × *P. nigra*, transcriptome and small RNA sequencing were employed to determine the expression profile of miRNAs and their targets in response to cold. Clusters of differentially expressed genes under cold treatment in *Populus simonii* × *P. nigra* were identified to be related various pathways which may be response to cold tolerance in *Populus simonii* × *P. nigra.* The data of miRNAs and transcriptome that we obtained should help reveal the molecular mechanisms responsible for cold tolerance.

## 2. Methods

### 2.1. Plant Materials and Treatment

Twelve overwintering branchlets of *Populus simonii* × *P. nigra* were collected from the campus of Northeast Forestry University, Harbin, China, (126°37′ E, 45°42′ N) and treated with hydroponic culture under a constant relative humidity of 50–60% and a 14 h light/10 h dark cycle at 24 °C ± 2 °C. Two days later, the branchlets were divided into two groups and subjected to cold (−4 °C) or not for 8 h with light (15 W/m^2^). Then, three leaf buds were sampled from each branchlet of two groups (total 18 leaf buds from each cold treated and untreated samples) and immediately frozen in liquid nitrogen for RNA preparation. For real time PCR analysis, twelve samples were divided into two groups treated under the same conditions in triplicate and then the total RNA of the leaf buds (six buds for each replication of cold treated and untreated samples) was prepared for experiment 2.6. Total RNA extraction and RNA library construction for sequencing.

### 2.2. Total RNA Extraction and RNA Library Construction for Sequencing

Total RNA was isolated from the cold-treated and untreated *P. simonii* × *P. nigra* plantlets using Universal Plant Total RNA Extraction Kit (Bioteke, Beijing, China) following the manufacturer’s protocol. Total RNA was quantified and assessed for quality using a NanoPhotometer (IMPLEN, Westlake Village, CA, USA) and Agilent 2100 (Agilent Technologies, Folsom, CA, USA). Cold-treated (CTD) and untreated (UD) sRNA libraries and cDNA libraries were constructed using Small RNA Sample Pre Kit (Illumina, San Diego, CA, USA) and NEBNext^®^ Ultra™ RNA Library Prep Kit for Illumina^®^ (NEB, Ipswich, MA, USA) following the manufacturer’s recommendations. Then, the sRNA libraries and cDNA libraries were sequenced on an Illumina Hiseq platform (Novogene, Beijing, China).

### 2.3. Identification and Differential Expression Analysis of miRNAs

The raw sequences were firstly processed to filter out the 5′ adapter (5′-GTTCAGAGTTCTACAGTCCGACGATC-3′) and 3′ adapter (5′-AGATCGGAAGAGCACACGTCT-3′) sequences, N% > 10%, low-quality as well as polyA/T/G/C sequences, and to obtain the final clean reads. Then, the small RNA sequences with 18–30 nt in length were subjected to *Populous tomentosa* mRNAs, Rfam (http://rfam.xfam.org/), and NCBI GenBank databases (http://ftp.ncbi.nlm.nih.gov) to identify mRNA, rRNA, tRNA, snRNA, snoRNA, and repeat sequences. Finally, the remaining unique sequences were analyzed by BLAST against miRBase (Release 21, http://www.mirbase.org/) and sequences with identical or related (1–3 bases mismatch) sequences from mature miRNAs were identified as known miRNAs [24]. Potentially novel miRNAs were identified by folding the flanking genome sequence of unique small RNAs using MIREAP https://sourceforge.net/projects/mireap/. The RNA secondary structure of the predicted miRNAs was checked using Mfold http://www.bioinfo.rpi.edu/applications/mfold/cgi-bin/rna-form1.cgi [25]. The potential targets of conserved and novel miRNAs were predicted using the psRobot program [26] http://omicslab.genetics.ac.cn/psRobot/ with default parameters. The miRNA expression levels between the two libraries (CTD and UD) were analyzed by the ratio of normalized read count in CTD and normalized read count in UD. The read counts from each library were normalized to transcripts per million reads (TPM) as follows: Normalized expression = (actual miRNA count/total count of clean reads) × 1,000,000. Then DEGseq was used to differential expression analysis and the differential expression miRNAs were screened with *q* value (*q* value < 0.01) and foldchange (|log_2_(foldchange)| > 1). Hierarchical clustering of differentially expressed miRNAs was performed using the Cluster (v3.0)/Treeview software (v3.0) (http://bonsai.hgc.jp/~mdehoon/software/cluster/software.htm). The TPM data adjusted by log_10_ (TPM + 1) was used as the value of hierarchical clustering.

### 2.4. Analysis of Differential Expression Genes Based on Transcriptome Sequencing

Raw data (raw reads) of fastq format were firstly processed to remove adaptor sequence, low-quality reads (more than 20% nucleotides with quality value ≤10), and shorter reads (<50 bp) and to obtain clean reads. After that, Q20, Q30, GC-content, and sequence duplication level of the clean data were calculated. Then contigs were assembled and obtained by using Trinity [27] with min_kmer_cov set to two by default. Next, the reads were mapped back to contigs to obtain longer sequences (transcripts), and the main transcript was defined as a unigene. After that, ORFs were predicted for all unigenes using Getorf (http://emboss.sourceforge.net/apps/cvs/emboss/apps/getorf.html) and Bowtie program was used to map the reads from two libraries to unigenes [28]. Differential expression analysis of CTD and UD data was performed using the DEGSeq R package (1.12.0) [29] based on the negative binomial distribution [30]. The resulting P values were adjusted using the Benjamini and Hochberg’s approach for controlling the false discovery rate [31]. Genes with *q* value < 0.005 & |log_2_(foldchange)| > 1 found by DEGSeq were assigned as differentially expressed.

### 2.5. GO and KEGG Pathway Enrichment Analysis

Gene Ontology (GO) enrichment analysis of the differentially expressed genes (DEGs) was implemented by the GOseq R packages based Wallenius noncentral hyper-geometric distribution [32], which can adjust for gene length bias in DEGs. KEGG [33] was utilized for understanding high-level functions and utilities of the biological system (http://www.genome.jp/kegg/). We used KOBAS [34] software to test the statistical enrichment of differential expression genes in KEGG pathways.

### 2.6. Quantitative Real-Time PCR Analysis

Total RNA was obtained from cold-treated and untreated *P. simonii* × *P. nigra* cuttages. For cDNA synthesis, 1 μg of total RNA of each sample was reverse transcribed with stem-loop primers (miRNA expression analysis) or oligo dT primers (target gene expression analysis) using the PrimeScript Reagent Kit with gDNA Eraser (Takara, Dalian, China). Several cold-responsive miRNAs and genes were validated and quantified using qRT-PCR. Primers were designed according to the miRNA sequences (Table S1) and assembly data using the online Primer BLAST program (https://www.ncbi.nlm.nih.gov/tools/primer-blast) (Table S2). The qRT-PCR was performed using the LightCycler 480 SYBR GREEN I Master (Roche Applied Systems, Mannheim, Germany) and ROCHE LightCycler 480 real-time system (Roche Applied Systems). Each reaction with a 20 μL volume was carried out containing 10 μL of PCR Master Mix, 1 μL of the first-strand cDNA, and 0.5 μM of each primer. The PCR amplification program consisted of 95 °C for 10 min, followed by 40 cycles of 95 °C for 15 s and 60 °C for 1 min. A thermal denaturing cycle of 95 °C for 1 min and 65 °C for 30 s was applied to determine the dissociation curves for verifying the specificity of the PCR amplifications. Three biological replicates and three technical replicates were performed for each of the analyzed genes. The actin gene was used as an internal reference for normalization. Relative transcript levels of each gene were calculated with the comparative cycle threshold (2-dCt) method [35].

## 3. Results

### 3.1. Identification of miRNAs in Populus simonii × P. nigra

**Figure 1:**
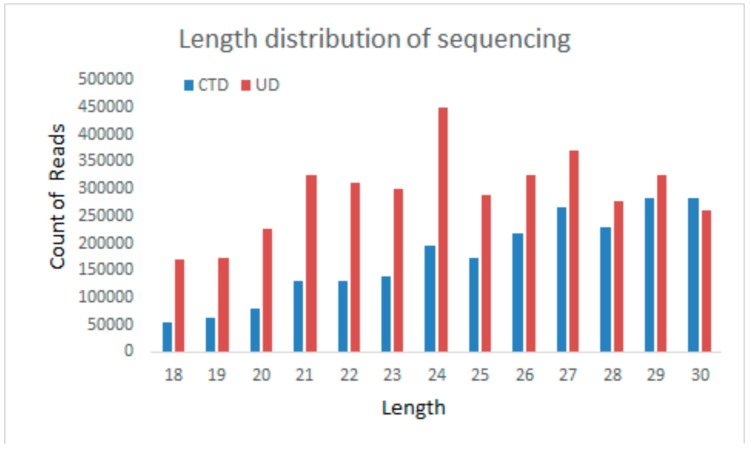
Total sRNA read length distribution of the sequencing libraries (Cold treated, CTD; Untreated, UD).

**Figure 2:**
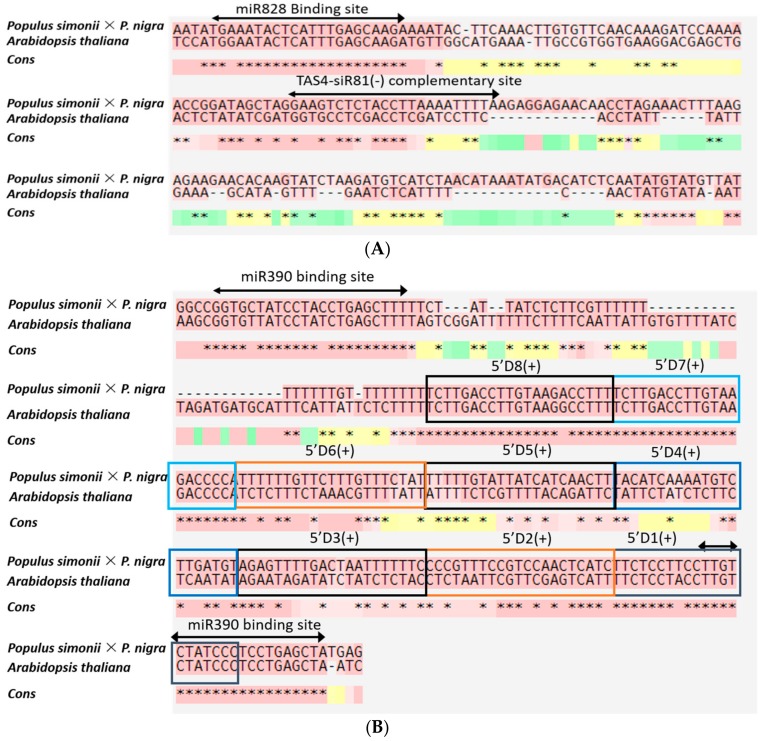
Sequence alignment of putative TAS3 and TAS4 paralogs in *Populous simonii* × *P. nigra* and *Arabidopsis thaliana* (Black arrow shows the complementary site of *TAS4-siR81*) (**A**) and the binding site of miR390 (**B**) miR828. Boxes indicate phased ta-siRNA and sequences of 5′D1(+) to 5′D8(+) that are proved in *Arabidopsis*.

Two RNA-seq libraries from cold-treated (CTD) and untreated (UD) plantlets were constructed for high-throughput sequencing. After discarding the low-quality sequences and adapter sequences, 6,356,896 (CTD) and 6,125,123 (UD) clean reads were obtained. Of 18–30 nt sRNAs, 24 nt miRNAs were the most abundant in CTD and UD samples (Figure 1) which is consistent with the distribution patterns of sRNAs in *Brassica napus* and tea plant [15,36]. Also, 10,089 (CTD) and 45,241 (UD) small RNA reads were found to be similar to known miRNAs after comparing with mature miRNA sequence or miRNA precursor in miRBase 21.0. The rest of the sequences were rRNA, snRNA, snoRNA, novel miRNA, or ta-siRNA (Table 1). Among the classified sRNAs, the proportion of sRNAs reduced in CTD compared to sRNAs in UD sample implying that miRNAs might be involved in the regulation of cold tolerance. Except known miRNAs, the function of unknown cold responsive sRNAs remained to be identified. A total of 87 (UD) and 63 (CTD) conserved miRNAs belonging to 34 miRNA families were identified in the two libraries (Table S3). Among these miRNA families, the miR166 family is the most abundant, followed by miR159 and miR472 family in both libraries. Other miRNA families such as miR482, miR319, and miR398 were also detected at high copy number. According to the predicting hairpin structures, 16 novel miRNAs candidates with hairpin sequence were identified (Table S4). Additionally, we identified the candidate phased-siRNA in *Populus simonii* × *P. nigra* from CTD and UD sRNA libraries. In total, 31 phased-siRNA were obtained (reads ≥4) and mainly derived from four predicted phased loci (Table S5). The Cluster-12280.0 loci overlapped by siRNAs is homologous to TAS4 which is predicted to be triggered by miR828, and Cluster-16493.19914 loci is similar to TAS3 which is predicted to be bound by miR390 and initiated phasiRNA (Figure 2). Among the candidate ta-siRNA, most were identified from UD sRNA libraries.

### 3.2. Differential Analysis of Candidate miRNAs Involved In Cold Tolerance in Populus simonii × P. nigra

**Figure 3:**
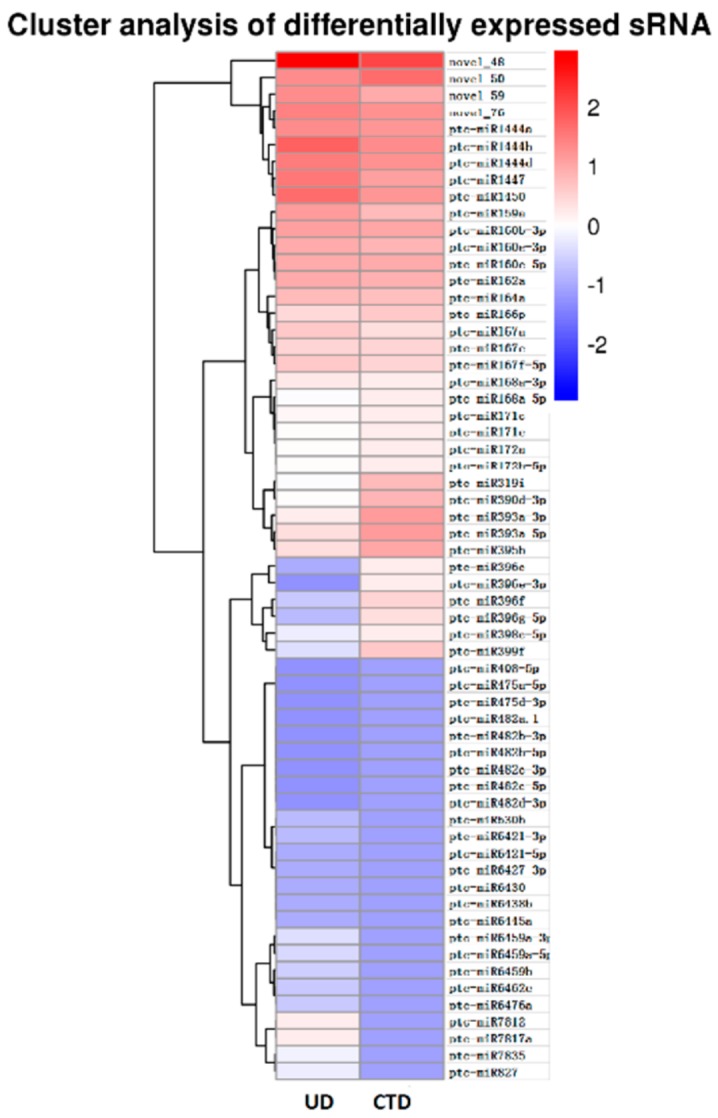
Hierarchical clustering of differentially expressed sRNAs in *Populus simonii* × *P. nigra* under cold-treated and untreated conditions.

To accurately measure the abundance of miRNA, the reads of each miRNA was normalized to generate RPM (reads per million) and analyzed by DEGseq [29]. The threshold of log_2_ Ratio ≥1 was applied to identify the differential expression miRNAs. In total, 61 miRNAs including 57 known miRNAs and four novel miRNAs were significantly up- or downregulated under cold-treated or untreated conditions (Figure 3). Among the sRNAs detected in both CTD and UD libraries, 11 miRNAs, including miR164a, miR168a-5p, miR160e-5p, and miR482d-3p, were upregulated with the treatment of cold, while 50 miRNAs such as miR159a, miR1447, miR167e, miR168a-3p, miR1450, and miR162a were downregulated after cold treatment. Also, the greatest changes in abundance were miR171e and miR482d-3p, which showed more than four-fold differences in expression with cold treatment compared to without cold treatment. In addition, 25 miRNAs, including miR399f, miR6427-3p, miR160b-3p, miR482b-3p, and miR7817a, were only detected in the UD library, but certain miRNAs such as miR171c, miR172h-5p, miR390d-3p, miR393a-3p, miR482b-5b, and miR6421 were transcribed at low levels (Table S6).

### 3.3. Identification of Candidate Cold-Responsive Genes by Transcriptome Analysis

**Figure 4:**
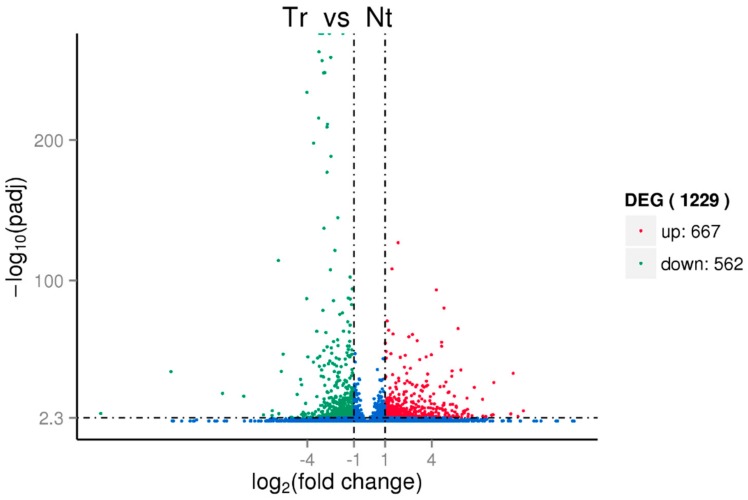
Differential expression genes (DEGs) identified in *Populus simonii* × *P. nigra* under cold treatment and untreated conditions.

The cold-responsive differential expression genes (DEGs) were also analyzed and identified through sequencing CTD and UD plantlet cDNA libraries. After removing low-quality reads and adapter sequences, 54,357,974 and 48,912,024 clean reads were obtained from the UD and CTD raw data separately. The Trinity program [27] was used to assemble transcripts and among 178,416 transcripts, 92,755 transcripts were annotated to be encoding genes (Table 2). Among the databases of NR, NT, PFAM, GO, and KOG, 78,569 (84.7%) genes could be annotated in NT and 63,389 (68.34%) in NR, and only about 50% (45,817) genes could be annotated by GO (Figure S1). In addition, most of genes had significant matches with *Populus trichocarpa* (60.4%) and *Populus euphratica* (25.4%) (Figure S2). The FPKM (expected number of fragments per kilobase of transcript sequence per million base pairs sequenced) was calculated and only those genes that had FPKM values > 0.3 were kept for analysis. After being normalized by TMM, a total of 1229 DEGs were detected by DEGseq analysis (*q* value < 0.005 & |log_2_ (fold change)| > 1) including 667 upregulated and 562 downregulated genes under cold-induced conditions (Figure 4 and Table S7). Differential expression analysis showed that during the cold treatment, many significantly differentially expressed genes are involved in the physiological and biochemical process of cold response. Further analysis by GO showed that among the 45 subsets of GO categories, the functional categories of metabolic process, carbohydrate metabolic process, oxidation-reduction process and oxidoreductase activity, metal ion binding, and cation binding are predominant (Figure S3). The cluster of GO: 0009409 represents response to cold and had four genes (Cluster-16493.37648, Cluster-16493.20026, Cluster-16493.34543, Cluster-16493.59475) upregulated and four genes (Cluster-16493.31741, Cluster-16493.32925, Cluster-16493.33751, Cluster-16493.32107) downregulated (Table S8). Among the annotated differential expression genes are Cluster-16493.58443 (putative dehydration-responsive element-binding protein 2C, DREB2C), Cluster-16493.25004 (putative DREB2A), Cluster-16493.44317 (putative DREB3), Cluster-16493.25005 (putative DREB2H) which contains a single APETALA2/Ethylene responsive element-binding factor (AP2/ERF) domain and can bind to the CRT/DRE (C-repeat/Dehydration Responsive Element) DNA element of many cold-responsive (COR) genes. Cluster-16493.27238 is annotated as a cold-shock DNA-binding family protein and transcription factor such as leucine zipper protein (Cluster-16493.40514, Cluster-16493.27643), MYB-like transcription factor (Cluster-16493.30912, Cluster-16493.18975), WRKY transcription factor (Cluster-16493.45530, Cluster-16493.18576), ethylene-responsive transcription factor (Cluster-16493.20988, Cluster-16493.19581), MADS-box transcription factor (Cluster-16493.34387, Cluster-16493.40727), bHLH transcription factor (Cluster-16493.25125), bZIP transcription factor (Cluster-16493.29872, Cluster-16493.39911) have been identified (Table S9).

### 3.4. Regulation Analysis of miRNAs via Prediction of Target mRNA

To identify the targets of differentially expressed miRNAs, 61 miRNAs were searched against the DEGs of *Populus simonii* × *P. nigra* using psROBOT (http://omicslab.genetics.ac.cn/psRobot/) and 988 candidate targets were predicted (Table S10), including putative transcription factor RAV1 (Cluster-16493.60622), MYB4 (Cluster-16493.7466), MYB86 (Cluster-4772.0), MYB46 (Cluster-8733.0), MYB104 (Cluster-15451.2), DOF3.7 (Cluster-18908.2), RAV1 (Cluster-16493.20287), AP2/ERF, and B3 domain-containing transcription factor (Cluster-16493.10150, Cluster-16493.60622), CRF3 (Cluster-16493.14255) and WRKY16 (Cluster-16493.22410, Cluster-16493.39399). These identified miRNAs and target genes may have important roles in resistance to cold treatment. Among these miRNA–target pair genes, the expression patterns between ptc-miR319i, ptc-miR7812, ptc-miR172a, ptc-miR6462e, ptc-miR1444d, ptc-novel_48 miRNA, and their targets were complementary. The target genes were annotated to encode heat shock protein (Cluster-16493.44708, Cluster-16493.49274), bZIP transcription factor (Cluster-16493.29872), endo-glucanase 2 family protein (Cluster-16493.45983), polyphenol oxidase (Cluster-16493.41325), and coatomer subunit alpha-1 (Cluster-16493.31604) and they were all upregulated by cold treatment (Table 3). The sRNA and transcriptome sequences were deposited in the Sequence Read Archive (SRA) of the National Center for Biotechnology Information (NCBI) (PRJNA513148).

### 3.5. Expression Analysis of miRNAs by qRT-PCR

**Figure 5:**
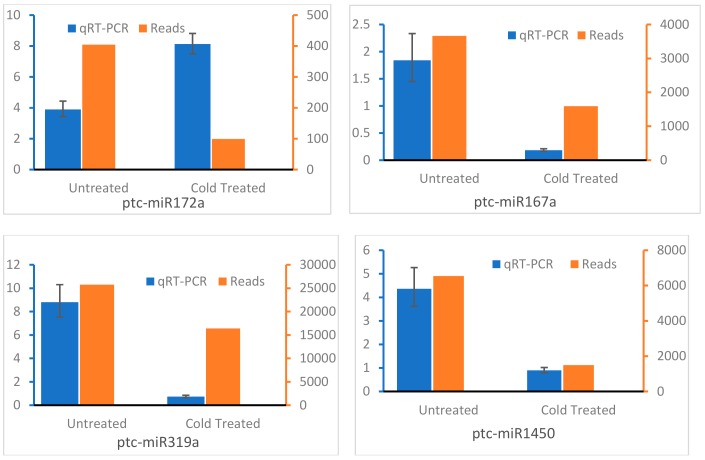
Comparing qRT-PCR data to reads analysis of candidate miRNAs in shoot of *Populus simonii* × *P. nigra* with and without cold treatment.

**Figure 6:**
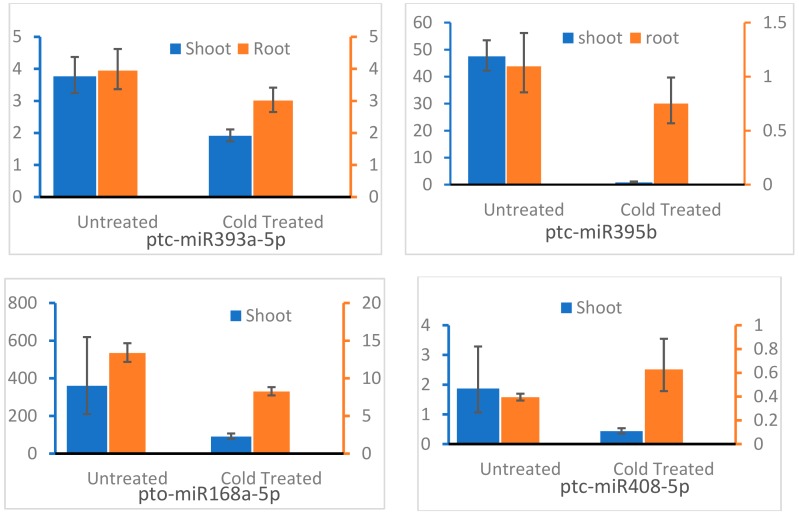
Relative expression analysis of candidate miRNAs in shoot and root of *Populus simonii* × *P. nigra* with and without cold treatment by real time PCR.

To validate the obtained differentially expressed miRNAs in response to cold, eight miRNAs were selected for further analysis by stem-loop qRT-PCR. Our result showed that miR167a, miR1450, miR319a, miR395b, miR393a-5p, miR408-5p, and miR168a-5p were downregulated after cold treatment (Figure 5), whereas transcription level of miR172a increased after cold treatment. The expression of most detected miRNAs was consistent with those by high throughput sequencing, except for miR172a and miR168a-5p. However, the transcription level of miR168a-5p was higher in both CTD and UD plantlets than that of other detected miRNAs, which was consistent with high throughput detection result. We also detected the expression of miR395b, miR393a-5p, miR408-5p, and miR168a-5p in CTD and UD roots and the results indicated that the expression of miR395b, miR393a-5p, and miR168a-5p decreased while the transcription level of miR408-5p was high in roots of cold-treated plants (Figure 6).

### 3.6. qRT-PCR Validation of the Transcription Level of Target Genes

**Figure 7:**
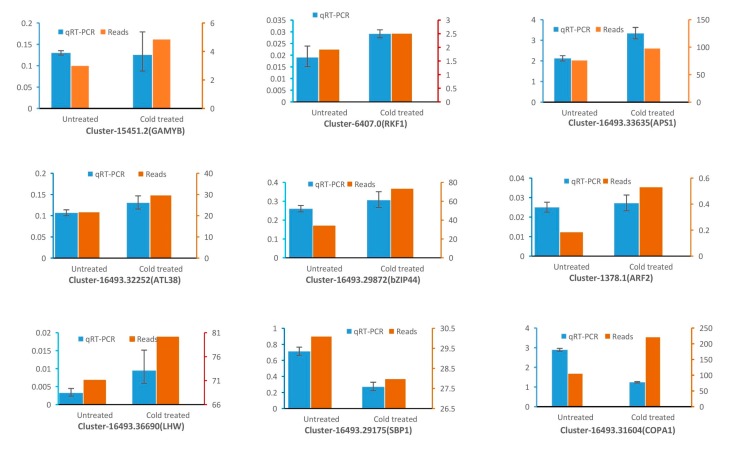
qRT-PCR expression levels of candidate targets detected in shoot of *Populus simonii* × *P. nigra* with and without cold treatment.

Through high throughput sequencing, many candidate genes have been screened from cold-treated and untreated plantlets of *Populus simonii* × *P. nigra.* To validate the results obtained by high-throughput sequencing, the transcription level of nine genes including Cluster-15451.2, Cluster-6407.0, Cluster-16493.33635, Cluster-16493.29175, Cluster-16493.29872, Cluster-1378.1, Cluster-16493.36690, Cluster-16493.32252, and Cluster-16493.31604 were confirmed by real time PCR. Our results showed that eight of the nine detected genes (except Cluster-16493.31604) had the same expression patterns with transcriptome sequencing (Figure 7) in CTD and UD plantlets. The expression of Cluster-15451.2 (annotated transcription factor GAMYB), Cluster-6407.0 (annotated LRR receptor-like serine/threonine-protein kinase RFK1), Cluster-16493.33635 (annotated ATP sulfurylase 1, APS1), Cluster-16493.32252 (RING-H2 finger protein ATL38), Cluster-16493.29872 (annotated bZIP transcription factor 44, bZIP44), Cluster-1378.1, and Cluster-16493.36690 (annotated transcription factor LHW) were upregulated under the cold treatment, whereas the transcription level of Cluster-16493.29175 (annotated squamosa promoter-binding-like protein 1, SBP1) and Cluster-16493.31604 decreased after cold treatment. The most abundant transcription level of these detected genes was Cluster-16493.31604 which was annotated coatomer subunit alpha-1-like isoform X1 (COPA1) and the least expression was Cluster-1378.1 which was annotated auxin response factor 2 (ARF2). In addition, transcription factor GAMYB was predicted to have the binding sites of ptc-miR319i, ptc-miR319a, ptc-miR319e, novel_63, ptc-miR159a, and ptc-miR159d. Also, the RFK1 (with binding site of ptc-miR164a), APS1 (with binding site of ptc-miR395b), SBP1 (with binding site of ptc-miR167a), bZIP44 (with binding site of ptc-miR172), LHW (with binding site of ptc-miR395b), ATL38 (with binding site of ptc-miR393a-5p), COPA1 (with binding site of ptc-miR482a.1, ptc-miR168a-5p, novel_48), ARF2 (with binding site of ptc-miR319a) were all predicted to be regulated by isolated miRNAs. Moreover, the expression patterns of ptc-miR395b with its candidate target LHW, and ptc-miR393a-5p with its candidate target ATL4, are opposite in CTD and UD plantlets (Figure 5, Figure 6 and Figure 7).

## 4. Discussion

In plants, cold injury severely affects geographic distribution. Recent reports have suggested that miRNAs play important roles in various abiotic response during development of plants through negatively controlling the expression of their target gene [2,37,38]. Although many genes have been identified in *Populus* under cold treatment [2,13], few studies have reported on *Populus simonii* × *P. nigra*, which is widely distributed in Northeast China. Further study is needed to elucidate the functions of miRNAs at a genome-wide level in response to cold tolerance. There is always a cold spell at the end of April and early May in which the temperature can drop below zero degree with snow in Heilongjiang area of China. Through cold treatment, we found plants treated with −4 °C for 12 h show obvious damage by freezing and can recover normal growth, but plants treated with −8 °C for 12 h cannot grow normally and plants treated with −4 °C for 4 h show light damage (Figure S4). Then, we used high throughput sequencing to analyze the transcription level of miRNAs and transcriptome in *Populus simonii* × *P. nigra* with or without treatment with −4 °C for 8 h. The results showed that the expression of most conserved miRNAs decreased and the transcription level of their candidate targets significantly increased in response to cold treatment, suggesting that cold induction lead to increased cold-responsive gene expression through decreasing the level of miRNAs. Thus, genome-wide analysis of miRNAs and targets response to cold can provide crucial information for our understanding of the molecular regulation of cold tolerance in *Populus simonii* × *P. nigra*.

### 4.1. Cold-Responsive miRNAs in Populus simonii × P. nigra

In our study, the transcription levels of miRNAs were analyzed in *Populus simonii* × *P. nigra* with and without cold treatment and the results showed that the expression of about 70% of miRNAs was affected by cold treatment. Among them, about 80% of miRNAs were downregulated, indicating that decreased expression of miRNAs might play more crucial roles than increased expressions of miRNAs in *Populus simonii* × *P. nigra.* In *Populus tomentosa*, downregulated miRNAs were more common than upregulated miRNAs in response to cold treatment [2]. Previous research indicated that psu-miR475 in *Populous suaveolens* [39], miR160, miR168, miR390, and miR396 in *Populus trichocarpa* [40], and pto-miR171, pto-miR319, and pto-miR395 in *Populus tomentosa* [2] respond to cold. Similarly, in the present study, these miRNAs showed obvious changes under cold treatment. Additionally, conserved miRNAs, such as ptc-miR1450, ptc-miR159a, ptc-miR164a, ptc-miR167a, ptc-miR172a, and candidate novel ptc-novel_48, ptc-novel_50, ptc-novel_59, and ptc-novel_76, were differentially expressed under cold treatment. These obtained miRNAs might downregulate the expression of their target genes which encode regulatory and functional proteins involved in cold tolerance.

Phased, secondary small interfering RNA (phasiRNA) are derived from phasiRNA-producing loci which were originally discovered in *Arabidopsis* and were also called trans-acting small interfering RNA (ta-siRNA) [41]. Four known TAS genes (TAS1 and TAS2 are targeted by miR173, and TAS3, TAS4 are targeted by miR390 and miR828 separately) are conserved in plants and their functions relate to each target of ta-siRNA and the expression pattern of targets [41,42]. Our results showed the siRNA Nt_403935_×34, with a similar sequence to 5′ D8 (+) originated from Cluster-16493.19914, was predicted to be ptc-TAS3 loci and was bound by ptc-miR390. The targets of siRNA (Nt_403935_×34) were predicted to be auxin response factors and their transcription level increased in CTD plantlets. Also, miR390-TAS3 tasiRNA-ARF2/3/4 has been reported to be integrated with auxin signaling to regulate lateral root growth of *Arabidopsis* [43]. This implied that miR390-TAS3 tasiRNA-ARF2/3/4 might also be involved in the process of cold resistance of *Populus simonii* × *P. nigra.* TAS4 loci, where the siRNAs were triggered by miR828 and targeted a set of MYB transcription factors [44] was also be predicted, but ptc-miR828 was not detected in either CTD or UD plantlets of *Populus simonii* × *P. nigra.* We postulated that the reason for undetected ptc-miR828 might be related to the low transcription level of ptc-miR828 verified by stem-loop RT-PCR according to the sequence of pto-miR828a in *Populus tomentosa*.

The predicted targets of differentially expressed miRNAs identified in our research have also been reported to be involved in cold tolerance. Our results showed the target gene of cold-responsive ptc-miR164a was LRR (leucine-rich repeat) receptor kinase, which is reported to be cold-inducible and has Ser/Thr protein kinase activity [45]. The conserved miR160 with potential target of ARF (auxin response factor) also showed a cold-resistance response in wheat [20]. The cold responsive miR167 has been identified to have LTR (low temperature response) element in the upstream regions [46] and candidate low temperature-induced protein was also predicted in our research. Other targets of miR167, such as ARF and SPL12 (Squamosa promoter-binding-like protein 12) were also predicted and reported to response to cold treatment [17] or thermo tolerance [47]. DnaJ, the candidate target of miR162a was also reported to contribute to maintenance of photosystem II under chilling induction [48]. In addition, Myb and TCP, candidate targets of ptc-miR319, have also been reported to be subjected to cold resistance in sugarcane [49]. Our results showed that cold induced the changes in transcription level of ptc-miRNAs and therefore led to the expression of target genes involved in cold resistance. Certainly, many of the cold responsive miRNAs have also been reported to be involved in plant growth and development, such as miR159 in growth and programmed cell death [50], miR172 in floret development [51], and miR319 in leaf development [52]. These results suggest that miRNAs have complex and widely regulatory roles in various plant stress and growth responses.

### 4.2. Differentially Expressed Transcripts under Cold Treatment

The transcriptome analysis showed many candidate genes differentially expressed in CTD and UD plantlets of *Populus simonii* × *P. nigra* which might be cold responsive genes. The functions of orthologs for these candidate genes, especially those encoded transcription factors involved in cold tolerance, have been clarified in other plants. DREB, the orthologs of which have been identified from *Populus simonii* × *P. nigra* in our research, is well known to be involved in abiotic stress. For example, AaDREB1, isolated from cold tolerant plant *Adonis amurensis*, showed enhanced tolerance to low temperature in transgeneic *Arabidopsis* and rice [53]. DREB/CBF (C-repeat binding factor) can recognize and bind to the CRT/DRE (C-repeat/Dehydration Responsive Element) motif to regulate cold-responsive (COR) genes [54]. The orthologs of leucine zipper protein (Cluster-16493.40514, Cluster-16493.27643) were also identified in *Populus simonii* × *P. nigra* and leucine zipper protein was reported to interact with CBF1B and involved in low temperature response in hot pepper [55]. MYB-like transcription factor (Cluster-16493.30912, Cluster-16493.18975) belongs to the R2R3-MYB family and recently MdMYB23 has been reported to be involved in cold tolerance in apple [56]. In rice, WRKY71 had positive function in cold tolerance by regulating downstream target genes [57] and Cluster-16493.45530 and Cluster-16493.18576 were also annotated to be WRKY family genes. MfERF1, one of ethylene-responsive transcription factor (ERF) was isolated from *Medicago falcata* and conferred cold tolerance [58] and the orthologs of ethylene-responsive transcription factor (Cluster-16493.20988, Cluster-16493.19581) were also isolated in our results. Moreover, the MADS genes [59], bHLH [60], bZIP [61], were also reported to be involved in cold resistance.

### 4.3. miRNAs May Be Involved in Cold Tolerance by Negatively Regulating Target mRNAs

**Figure 8:**
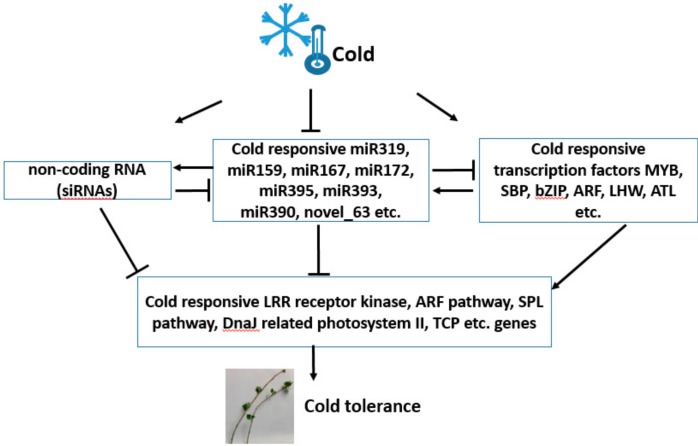
A putative cold responsive regulation model of sRNAs and their targets in *Populus simonii* × *P. nigra.*

The downregulated miRNAs in response to cold treatment in *Populus simonii* × *P. nigra* would positively regulate the expression of their targets for cold resistance. Integrated analysis of miRNAs and their targets expression helps to reveal the regulatory pathways of functional miRNA–mRNA modules (Figure 8). Based on data analysis, the putative cold responsive regulation model showed sophisticated up- and downregulated network. Firstly, the cold signal was perceived and transmitted to transcription factor genes such as MYB, SBP, bZIP, ARF, LHW, ATL, MIR genes such as miR319, miR159, miR167, miR172, miR395, miR393, miR390, novel_63, and ncRNA genes which can be targeted by miRNA for generating siRNAs. The expression of putative MIR showed downregulation, whereas the expression of candidate transcription factor genes were upregulated. Secondly, the negative regulation between miRNAs and their candidate targets, such as miR390 and noncoding RNA TAS3, miR319, and MYB led to the expression of cold responsive transcription factors and accumulation of cold responsive metabolic pathway genes. Thirdly, the metabolic genes related to the LRR receptor kinase pathway, ARF pathway, SPL pathway, and DnaJ-mediated photosystem II are taken as candidate genes involved in cold tolerance of *Populus simonii* × *P. nigra.* Although cold responsive miRNAs and candidate genes were isolated and characterized in *Populus simonii* × *P. nigra* and several other species, such as wheat [20], sugarcane [62], tea plant [15], soybean [63], and tomato plant [64], the mechanism of molecular regulation at the transcriptional and post-transcriptional level involved in the network of cold tolerance is still largely unknown. Further work is needed to elucidate the function of these important genes in cold resistance-related regulatory pathways.

## 5. Conclusions

Cold tolerance miRNAs and candidate target genes were identified through integrated sRNA and transcriptome analysis in cold treatment of *Populus simonii* × *P. nigra.* MIR genes such as miR319, miR159, miR167, miR172, miR395, miR393, miR390, and novel_63 and transcriptional factors including MYB, SBP, bZIP, ARF, LHW, and ATL showed differential expression and they might be the main contributors related to LRR receptor kinase, the ARF pathway, the SPL pathway, and DnaJ-related photosystem II involved in the cold tolerance of *Populus simonii* × *P. nigra.* These results not only increase our knowledge of sRNAs involved in the post-transcriptional regulation of cold tolerance, but also provide candidate genes for future functional analysis of the cold tolerance-related signaling pathways in *Populus simonii* × *P. nigra.*

## Figures and Tables

**Table 1:** Small RNAs obtained in cold-treated (CTD) and untreated (UD) sRNA libraries.

Sample	Total Reads	Clean Reads	Total sRNA	Mapped sRNA	rRNA	snRNA	snoRNA	Ta-siRNA	Known miRNA	Novel miRNA
Cold Treated	6,757,221	6,125,123	2,247,238	1,875,409	251,512	2012	19,535	235	10,089	118
Untreated	6,547,394	6,356,896	3,807,159	3,126,566	427,344	2570	34,948	2778	45,241	681

**Table 2:** RNA sequencing analysis in CTD and UD RNA libraries.

Sample	Raw Reads	Clean Reads	Q20 (%)	Q30 (%)	Number of Transcripts (>200 bp)	N50	N90	Number of Genes (>200 bp)	N50	N90
CTD	49,473,544	48,912,024	97.21	95.5	178,416	1347	266	92,755	1668	528
UD	55,000,000	54,357,974	97.17	95.46						

**Table 3:** The candidate differential targets of cold-responsive miRNAs in *Populus simonii* × *P. nigra.*

Target ID	Annotation	miRNA ID	Predicted Binding Site	Negative Regulation
Cluster-16493.31604	PREDICTED: coatomer subunit alpha-1-like isoform X1	novel_48	miRNA: 1 TGTGGGAATGAACATTATGAG 21 | : | || :|||||| | | : : | : || : Target: s312 ATACCTTTACTTGTGGTGCTT 292	yes
Cluster-16493.38601	Putative EG45-like domain containing protein 1	ptc-miR167e	miRNA: 1 TGAAGCTGCCAGCATGAT-CTG 21 |||| ||| : ||||| | *|| | **|| Target: 259 ACTTCGATGGTCGTCCTACAAC 238	no
Cluster-16493.31604	PREDICTED: coatomer subunit alpha-1-like isoform X1	ptc-miR168a-5p	miRNA: 1 TCGCTTGGTGCAGGTCGGGAA 21 |* ||||| | | : ||: : ||| | | || Target: 3706 ACCGAACCATGTTTAGCCCTT 3686	no
Cluster-16493.38601	Putative EG45-like domain containing protein 1	ptc-miR167f-5p	miRNA: 1 TGAAGCTGCCAGCATGATCTT 21 |||||| | : |||| | | *||| *|| Target: 259 ACTTCGATGGTCGTCCTACAA 239	no
Cluster-16493.38601	Putative EG45-like domain containing protein 1	ptc-miR167a	miRNA: 1 TGAAGCTGCCAGCATGATCTA 21 ||||| || : ||| ||| * |||* |* Target: 259 ACTTCGATGGTCGTCCTACAA 239	no
Cluster-16493.40057	LINE-1 retrotransposable element ORF2 protein	ptc-miR396e-3p	miRNA: 1 CTCAAGAAAGCTGTGGGAGA 20 * : *|| |||| |||||| : | | | | Target: 2923 CGCTTCTTTCGACACTCTCT 2904	no
Cluster-16493.31604	PREDICTED: coatomer subunit alpha-1-like isoform X1	ptc-miR482a.1	miRNA: 1 CCTACTCCTCCCATTCC 17 ||||* | |* | || || |: || Target: 1901 GGATAAGAAGGGTAGGG 1885	no
Cluster-16493.26584	Calcium-transporting ATPase 4	ptc-miR482a.1	miRNA: 1 CCTACTCCTCCCATTCC 17 *|| *| || || |* |||||| Target: 4880 TGAAGAGGAGTGTAAGG 4864	no
Cluster-16493.41325	Polyphenol oxidase, chloroplastic	ptc-miR1444d	miRNA: 1 CGAACGTTGACCGAATGT-GAA 21 || ||||| : |||||||||| * | * | Target: 85 GCTTGCAGCTGGCTTACACCCT 64	yes
Cluster-16493.29872	Basic leucine zipper 63	ptc-miR172a	miRNA: 1 AGAATCTTGATGATGCTGCAT 21 | : || | |||||| |* |||| |* * | Target: 1241 TTTTAGAACTACGACGACCAA 1221	yes
Cluster-16493.41106	Putative SWI/SNF-related matrix-associated actin-dependent regulator of chromatin subfamily A member 3-like 2	ptc-miR172a	miRNA: 1 AGAATCTTGATGATGCTGCAT 21 | || || |||| | |*|*| || * ||| Target: 3710 TCTTAGAACTAGTCCGA-GTA 3691	no
Cluster-16493.44708	Heat shock protein 90-1	ptc-miR319i	miRNA: 1 TTGGGCTGAAGGGAGCTCCC 20 ||| * ||: | | || : || || ||| * Target: 890 AACGCGGCTTCTCTCGAGGT 871	yes
Cluster-16493.49274	18.2 kDa class I heat shock family protein	ptc-miR7812	miRNA: 1 CTGTTATGAATTGATGGAGTG 21 * ||* ||| |||| || |||||*|| Target: 305 AACTATACTTAACTACCT-AC 286	yes
Cluster-16493.45983	endo-glucanase 2 family protein	ptc-miR6462e	miRNA: 1 TCTTATGCGTTTTTGTCTCT 20 |||| || ||| ||| : : : | |* *| Target: 590 AGAATACGCAAAGGTAG-TA 572	yes

## Supplementary Materials

The following are available online at https://www.mdpi.com/2073-4425/10/6/430/s1. The data sets supporting the results of this article are included with the manuscript and its additional files. The sRNA and transcriptome sequences were deposited in the Sequence Read Archive (SRA) of the National Center for Biotechnology Information (NCBI) (PRJNA513148).

## Abbreviations

AP2/ERF: APETALA2/Ethylene responsive element-binding factor; ARF: auxin response factor; miRNAs: microRNAs; CBF: C-repeat binding factor; COR: cold-regulated; CRT/DRE: C-repeat/Dehydration Responsive Element; CTD: cold treated; DCL: Dicer-Like; DEGs: differential expression genes; DREB2C: dehydration-responsive element-binding protein 2C; ERF: Ethylene-responsive transcription factor; FKPM: Fragments Per Kilobase of transcript sequence per Millions base pairs; RISC: RNA-induced silencing complex; ROS: reactive oxygen species; RPM: reads per million; SBP1: squamosa promoter-binding-like protein 1; UD: untreated.
