# Supplementary figures and images for: Genome-Wide Analysis of the miRNA–mRNAs Network Involved in Cold Tolerance in Populus simonii × P. nigra
Source: Genes (Basel). 2019 Jun 5;10(6):430. doi: 10.3390/genes10060430 (PMC6627750; doi:10.3390/genes10060430)

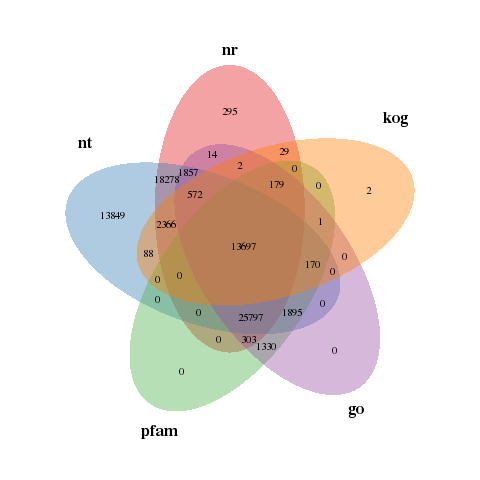

Supplement: Supplementary file 1 [file genes-10-00430-s001.zip › Figure S1.png]

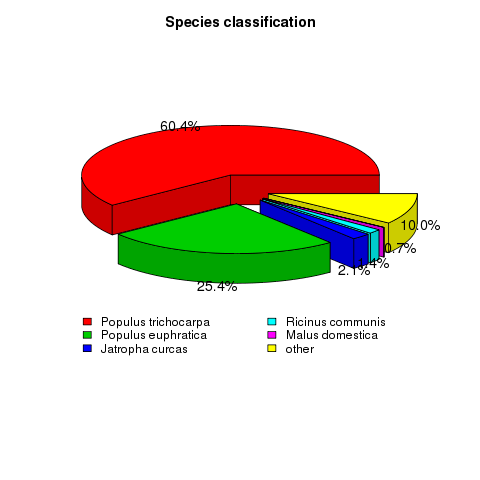

Supplement: Supplementary file 1 [file genes-10-00430-s001.zip › Figure S2.png]

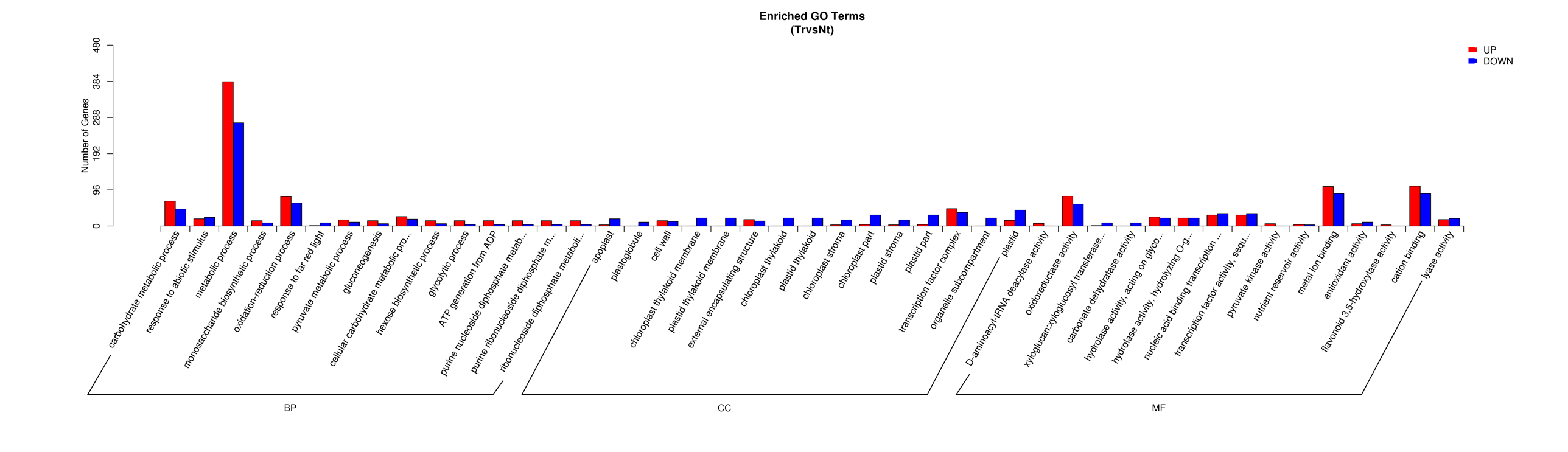

Supplement: Supplementary file 1 [file genes-10-00430-s001.zip › Figure S3.png]
