# Supplementary material for: Genome-Wide Analysis of the miRNA–mRNAs Network Involved in Cold Tolerance in Populus simonii × P. nigra
Source: Genes (Basel). 2019 Jun 5;10(6):430. doi: 10.3390/genes10060430 (PMC6627750; doi:10.3390/genes10060430)
Supplement: Supplementary file 1 [file genes-10-00430-s001.zip › Table S1.doc]

**Table S1** Primers for real-time reverse transcription-PCR validated miRNAs

| miRNA ID | Reverse transcription primer sequence | Forward primer sequence |
| --- | --- | --- |
| ptcrmiR167a | GTCGTATCCAGTGCAGGGTCCGAGGTATTCGCACTGGATACGACTAGATC | gacgcaTGAAGCTGCCAGCAT |
| ptcrmiR319a | GTCGTATCCAGTGCAGGGTCCGAGGTATTCGCACTGGATACGACGGGAGC | gacgcaTTGGgCTGAAGGGA |
| ptcrmiR395b | GTCGTATCCAGTGCAGGGTCCGAGGTATTCGCACTGGATACGACGAGTTC | gacgcaCTGAAGTGTTTGGGG |
| ptcrmiR1450 | GTCGTATCCAGTGCAGGGTCCGAGGTATTCGCACTGGATACGACTAACCT | gacgcaTTCAATGGCTCGGTC |
| ptcrmiR408-5p | GTCGTATCCAGTGCAGGGTCCGAGGTATTCGCACTGGATACGACCCATGC | gacgcaCGGGGAACAGGCAGA |
| ptcrmiR164a | GTCGTATCCAGTGCAGGGTCCGAGGTATTCGCACTGGATACGACTGCACG | gacgcaGGAGAAGCAGGGCA |
| ptcrmiR393a-5p | GTCGTATCCAGTGCAGGGTCCGAGGTATTCGCACTGGATACGACAGATCA | gacgcaTCCAAAGGGATCGCAT |
| ptcrmiR172a | GTCGTATCCAGTGCAGGGTCCGAGGTATTCGCACTGGATACGACATGCAG | gacgca AGAATCTTGATGATG |
| RT-reverse primer | CCAGTGCAGGGTCCGAGGTA |  |
| ptcActin | ACCAGAGTCCAACACAATACC | ACCTTCAACACTCCTGCTATG |
