# Supplementary material for: Genome-Wide Analysis of the miRNA–mRNAs Network Involved in Cold Tolerance in Populus simonii × P. nigra
Source: Genes (Basel). 2019 Jun 5;10(6):430. doi: 10.3390/genes10060430 (PMC6627750; doi:10.3390/genes10060430)
Supplement: Supplementary file 1 [file genes-10-00430-s001.zip › Table S2.doc]

**Table S2** Primers used in real-time PCR

| Primer name | Forward primer Sequence (5’-3’) | Reverse primer Sequence (5’-3’) |
| --- | --- | --- |
| Cluster-16493.40501 | AAGGAGTCGGCATGATGAGC | GTGGATGCAGACACCACTGA |
| Cluster-16493.29175 | GCAAGCCATGACGTTGAACA | TGTTGCGGCATACCAGACAT |
| Cluster-16493.29872 | CAAGCCAAATTGCCAGAATACCG | CTGTCTTGTTCAGGCGCAAT |
| Cluster-16493.31604 | TACGGCAGCTAACTTTGCCA | CTGCCCTTCATGGCTTGGTA |
| Cluster-1378.1 | GCGAAGCAAAGACGTCAGG | TGGGCATTGCCGTCTTACTA |
| Cluster-16493.28995 | CACAACTGACCGCGGATAAAC | GACTCACGGCATGGATTCCT |
| Cluster-6407.0 | AACTGGACTGGAGGGACCTA | AAGGTAGAGTTCACCCCTTCA |
| Cluster-16493.36690 | GGTTTGGAGGTCAGCGAGTT | AAGCGAACAGAGTCTACCCC |
| Cluster-16493.53626 | ACAGTGAGCATCTCCAGCAC | TTCAGTTCAGGTCCTCCCCA |
| Cluster-16493.32252 | CAGGCTAGTGCAAGCGCAAA | GGGGATGACTTGGTGATGTGA |
| Cluster-16493.33635 | TTCAACTCGCTCCGTTTGGA | TCCCCATGTTCTCGCAATCC |
| Cluster-15451.2 | ATGCGGTCAGATGGTCAGTC | TATGCTCGTCCATCTGGGGA |
| Cluster-16493.37673 | TCACGTGTTAACTTCAATTCCATT | CACTCGTCAAAGAGGCAATAACA |
| Cluster-16493.49135 | ATTTCGCCAACAACAACCCG | AATTCGAGTCCGAGAGCGAC |
| Cluster-16493.16428 | GTGCGTGCGCAATAAACCAT | GCCGCCTCCTCTAACATCTG |
